# Supplementary material for: A set of multi-entry identification keys to African frugivorous flies (Diptera, Tephritidae)
Source: Zookeys. 2014 Jul 24;(428):97–108. doi: 10.3897/zookeys.428.7366 (PMC4143993; doi:10.3897/zookeys.428.7366)
Supplement: Supplementary material 6 — Key to Ceratitis [file zookeys-428-097-s006.zip › SF6_ZooKeys_key to Ceratitis/key/SF6_ZooKeys_key to Ceratitis/Media/Html/Ceratitis contramedia .htm]

Ceratitis (Ceratalaspis) contramedia (Munro)


***Ceratitis*** **(*Ceratalaspis*)
*contramedia* (Munro)**

*Pardalaspis contramedia* Munro, 1937: 2.

Body
length: 5.14 (4.75-5.50) mm; wing length: 5.29 (4.20-6.75) mm.

Male

Head.
Antenna yellow-orange. Third antennal segment three times as long as second
segment. Arista bare. Ocellar bristles well developed but short, at most 1.5
times as long as ocellar triangle. Frons with short scattered hairs which
distinctly darker than frons; more flattened, not distinctly convex, in lateral
view distinctly projecting forwards at antennal implant. Chaetotaxy normal for
subgenus.

Thorax.
Postpronotum more or less concolorous with centre mesonotum, only slightly less
orange, unspotted. Ground colour of mesonotum pale with orange tinge.
Chaetotaxy normal for subgenus. Scapular setae pale. One anepisternal bristle.
Scutellum yellow with three apical black markings which are separated except
narrowly joined along apical margin; basally without dark spots, only slight
darker colouration. Subscutellum with two spots.

Legs
yellow; setation typical for subgenus, with mixed pale and darker hairs.
Posterior and posterodorsal rows on fore femur with pale bristles except apical
half of posterior row with dark bristles.

Wing
bands with yellow-brown markings; bands not very clear in some specimens
especially not discal and cubital bands. Banding, marginal band continuous;
cubital band free; medial band absent; crossvein r-m well before middle of
discal cell, usually at basal third. Crossvein dm-cu posteriorly more inwards
than anteriorly.

Abdomen.
Pale yellow or more brownish, with darker coloured spots (spots not as
outspoken as in *Pardalaspis* species). Otherwise setation and banding
typical for subgenus.

Female

As
male except for the following characters: Oviscape at least as long as
abdominal terga 3-6 combined.

(Description
after De Meyer, 1998)
